# Effects of exercise training on systemic arterial pulse wave velocity in postmenopausal women: an updated systematic review and meta-analysis of randomized controlled trials

**DOI:** 10.1186/s13102-025-01382-1

**Published:** 2025-11-11

**Authors:** Yu-Hong Li, Na Xu, Fei-Fei Ren, Su-Jie Mao, Wen-Sheng Zhou

**Affiliations:** 1 College of Physical Education, Jiangsu Second Normal University, 6 Xinhe West Road, Lishui District, Nanjing, 211200 China; 2 Department of Physical Education, Beijing Language and Culture University, Beijing, China; 3 Graduate Department, Harbin Sport University, Harbin, China

**Keywords:** Middle-aged and elderly women, Physical activity, Cardiovascular health, Arterial stiffness, Postmenopause

## Abstract

**Background:**

Postmenopausal women experience accelerated ageing of arterial vessels and increased cardiovascular disease risk. Exercise training, as a non-pharmacological intervention, holds great value in improving cardiovascular issues such as arterial stiffness.

**Objective:**

The present systematic review and meta-analysis aimed to: (1) synthesize current evidence on the efficacy of exercise in improving systemic arterial pulse wave velocity (SAPWV) in postmenopausal women; (2) clarify differential effects of exercise interventions on central versus peripheral PWV; and (3) quantify the statistical moderating effects of exercise protocol parameters and participant characteristics.

**Methods:**

Six electronic databases (EMBASE, EBSCOhost, Scopus, Web of Science, PubMed, Cochrane CENTRAL) were systematically searched up to August 7, 2024, and updated on April 4, 2025. Randomized controlled trials (RCTs) examining the effects of exercise training on PWV in postmenopausal women were included. A three-level meta-analysis was conducted using a random-effects model, as applied in R. Study quality was assessed with the Physiotherapy Evidence Database (PEDro) scale.

**Results:**

Nine studies (27 RCTs) were included. Compared with the control group, the overall effect of exercise on SAPWV reached only marginal significance [g = -1.07; 95% confidence intervals (CI) = -2.15, 0.00, *p* = 0.05], with the CI including zero—indicating that the true effect could be null or even negative. Additionally, exercise had significant improvements on SAPWV sub-indicators, including carotid-femoral PWV (cfPWV; g = -2.44; 95% CI = -3.94, -0.93) and central PWV (g = -1.57; 95% CI = -2.84, -0.30). The improvement in SAPWV was more pronounced when participants aged < 65 years (g = -1.52; 95% CI = -2.80, -0.24), exercising ≥ 3 times per week (g = -1.14; 95% CI = -2.25, -0.04), and engaging in aerobic exercise (g = -1.76, 95% CI = -3.13, -0.40). PWV sub-indicators, regional PWV, and exercise type moderated the effects of exercise training on SAPWV.

**Conclusion:**

The present study demonstrated that exercise training may improve arterial stiffness in postmenopausal women, with particularly pronounced effects on cfPWV and central PWV. Subgroup analysis further revealed that participants aged < 65 years, those exercising ≥ 3 times weekly, and those engaging in aerobic exercise interventions all demonstrated significant reductions in SAPWV. Although the overall effect of exercise on SAPWV reached only marginal significance, more pronounced effects were observed within specific populations and exercise modalities. Future large-sample studies are needed to validate these findings further.

**Supplementary Information:**

The online version contains supplementary material available at 10.1186/s13102-025-01382-1.

## Key points

- Exercise leads to particularly pronounced reductions in stiffness of central arteries, as evidenced by significant improvements in both carotid-femoral PWV and central PWV - indicators strongly linked to cardiovascular mortality.
- The greatest improvements in SAPWV occur in women aged < 65 years, those exercising ≥ 3 times per week, and those participating in aerobic exercise training, highlighting key moderating factors for efficacy.
- The overall effect analysis indicated a marginally significant effect of exercise on SAPWV in postmenopausal women, but this finding must be interpreted with caution due to high heterogeneity, publication bias, and the marginal nature of the statistical significance.

**Supplementary Information:**

The online version contains supplementary material available at 10.1186/s13102-025-01382-1.

## Introduction

Postmenopausal women exhibit accelerated arterial ageing and arterial stiffening [1, 2], with arterial stiffness significantly exceeding that in men after the age of 58 [3]. Arterial stiffness is a key factor contributing to the marked increase in cardiovascular risk among postmenopausal women, which can trigger a series of problems, including cardiac remodeling, ventricular hypertrophy, diastolic dysfunction, atrial fibrosis, and impaired coronary perfusion. Severe cases may lead to heart failure, intracerebral haemorrhage, and cognitive impairment [3–7]. Cardiovascular diseases, such as arterial stiffness, have become the leading cause of death in postmenopausal women aged 65–70 [8, 9]. Pulse wave velocity (PWV) is a crucial indicator for assessing arterial stiffness. Depending on the measurement site, it is classified into central and peripheral arteries, including indices such as carotid-femoral PWV (cfPWV), brachial-ankle pulse wave velocity (baPWV), aortic PWV, femoral-ankle pulse wave velocity (faPWV), heart-brachial pulse wave velocity (hbPWV), and leg PWV [10–12]. Studies have shown that each 1 m/s increase in PWV is associated with a 43% increase in all-cause mortality risk and a 54% increase in cardiovascular mortality risk [13].

Previous research has indicated that the increase in arterial stiffness is associated with the decline in estrogen levels after menopause [14]. Following menopause, the abrupt decline in estrogen impairs vascular endothelial repair and protection, resulting in endothelial cell damage. This alteration triggers a cascade of pathological responses: activation of inflammatory reactions attracts inflammatory cell aggregation and amplifies inflammatory factor release; enhanced oxidative stress concurrently stimulates smooth muscle cell proliferation. Simultaneously, significant blood pressure elevation and disrupted lipid metabolism occur. Particularly, the increase in LDL-C further leads to the accumulation of cholesterol within the vascular wall, ultimately resulting in endothelial dysfunction, accelerating the formation of atherosclerosis, and further contributing to increased arterial stiffness [14–17]. Hormone replacement therapy (HRT) is potentially beneficial for cardiovascular health in postmenopausal women. However, a large-scale cohort study involving over 900,000 individuals indicated that the risks of HRT outweigh its benefits, increasing the incidence of cardiovascular events including ischemic heart disease, ischemic stroke, cerebral infarction, and myocardial infarction [18], while also increasing risks for breast cancer and endometrial carcinoma [19–21]. Statins are utilized to treat arterial stiffness and reduce cholesterol levels, yet they pose potential adverse effects such as hepatic dysfunction, rhabdomyolysis, myasthenia, and gastrointestinal disturbances [22, 23].

Non-pharmacological interventions (e.g., exercise and physical activity) has been proved to promote overall health and delay the aging process [24, 25]. Moreover, exercise are widely recognized as safe strategies for ameliorating arterial stiffness in postmenopausal women. International health management guidelines consistently emphasize exercise as the primary intervention [26, 27]. Current evidence indicates that exercise may modulate lipid metabolism, enhance vascular elasticity, and slow arterial stiffening [15, 28–30]. However, the efficacy of exercise may be controversial. Ohta et al. (2012) and Wong et al. (2018) found that 12 weeks of aerobic exercise (such as stair climbing or step-ups) can reduce peripheral arterial baPWV [31, 32]. However, Kobayashi et al. (2022) found that aerobic exercise did not affect peripheral arterial baPWV [33]. In contrast, it produced marked improvements in central arterial cfPWV after only 8 weeks of intervention [33]. Regarding the efficacy of resistance exercise interventions, substantial controversy persists. Some studies have demonstrated that resistance exercise improves peripheral PWV (baPWV and leg PWV) [34, 35], while showing no significant effect on central PWV (aortic PWV and cfPWV) [34–36]. Moreover, additional research has confirmed that resistance exercise has no improvement in peripheral PWV (faPWV and baPWV) [36]. The different measurement sites of PWV may be the cause of the inconsistent findings. A systematic review study indicates exercise significantly reduces peripheral PWV baPWV by 0.69 m/s in postmenopausal women [37]. However, the effect on central PWV (such as aortic PWV and cfPWV) is uncertain. For example, resistance training can reduce arterial PWV (including cfPWV, baPWV, and faPWV) by 0.67 m/s in some meta-analyses [38], but other studies have not observed improvements in these indicators [28, 39]. The differential effects of aerobic and resistance training on SAPWV stem from distinct vascular mechanisms. Aerobic exercise enhances endothelial shear stress, upregulates endothelial nitric oxide synthase (eNOS) activity, increases nitric oxide (NO) bioavailability, and improves central arterial compliance—particularly in the aorta and carotid-femoral segment [40], leading to reduced stiffness and improved SAPWV. In contrast, resistance training induces transient blood pressure spikes and arterial wall stress [40, 41], potentially impairing vascular remodeling. While it may improve peripheral compliance through local adaptations, it lacks sustained hemodynamic stimulation for central arteries. Repeated high-pressure loading may even increase central arterial stiffness over time. These differences explain why aerobic exercise consistently benefits SAPWV and central PWV, whereas resistance training shows limited or no effect. These contradictions suggest that previous meta-analyses may not have adequately controlled for confounding factors such as exercise protocol characteristics and PWV measurement sites, or overlooked the moderating effects of participants’ baseline attributes (e.g., age, health status).

To our knowledge, this is the first systematic review and meta-analysis to apply a three-level random-effects model to account for dependency among multiple PWV outcomes within individual studies—a methodological advancement over traditional univariate meta-analyses. The present study provides an updated and more robust evidence base compared to previous reviews [35, 36], which either included fewer studies or failed to adequately account for the correlation between multiple PWV measures within the same study. Furthermore, we conducted subgroup analyses by anatomical site (central vs. peripheral PWV), exercise type, frequency, and duration, enabling a more nuanced understanding of how these factors moderate the effects of exercise on arterial stiffness. These methodological improvements enhance the analytical rigor and clinical relevance of our findings.

## Methods

### Design and eligibility criteria

The protocol for this systematic review was registered in the International Prospective Register of Systematic Reviews (PROSPERO registration number: CRD42024570021) on August 8, 2024. The study results were reported by the Preferred Reporting Items for Systematic Reviews and Meta-Analyses (PRISMA^®^) guidelines [42].

Eligible studies should meet the following Population, Intervention, Comparator, Outcome, and Study design (PICOS) criteria: 

*Participants*: Participants were defined as postmenopausal if the studies reported menopause status (e.g., ≥ 12 months of amenorrhea, serum FSH >30 IU/L, or clinical diagnosis by a physician). For studies that did not explicitly confirm menopause, a conservative lower age limit of ≥ 55 years was applied, based on population-based data indicating the average age of menopause onset is approximately 50–52 years [43–48], with potential variation of ± 1–2 years due to race, BMI, physical activity level, smoking status, and socioeconomic factors [43, 49]. This approach ensures inclusion of likely postmenopausal women while minimizing misclassification. To assess the robustness of our findings, a sensitivity analysis was conducted excluding studies where menopause was inferred solely from age (*n* = 3) [50–52], and the overall effect remained similar (g = −1.26, 95% CI = −2.89, 0.37, *p* = 0.124), suggesting the present results are robust to this criterion.

*Interventions*: Studies that used aerobic exercise or resistance training as the intervention.

*Comparator*: Studies that compared exercise (aerobic exercise, resistance training) with no exercise (no exercise, maintaining regular activity, and receiving health education courses, etc.).

*Primary outcome*: Studies that included PWV indicators such as cfPWV, baPWV, and faPWV.

*Types of studies*: Only RCTs published in English peer-reviewed journals were included.

Excluded studies were as follows: Ineligible subject age; non-aerobic exercise or non-resistance training; no control group or control group engaging in exercise; non-RCT; animal studies; unable to obtain full text or data; acute exercise intervention; conference abstracts or reviews.

### Literature search

A literature search was conducted in six electronic databases: EMBASE, EBSCOhost, Scopus, Web of Science, PubMed, and Cochrane CENTRAL. The search covered the period from the inception of each database to August 7, 2024, and was updated on April 8, 2025. The search terms included “Postmenopausal women”, “Middle-aged and elderly women”, “Aerobic exercise”, “Resistance exercise”, “Arterial stiffness”, and “Pulse wave velocity”. The detailed search strategy is provided in the Supplementary Material. The retrieved articles were downloaded into the EndNote reference management software. Two researchers (Y-HL and NX) independently screened the titles and abstracts of the articles according to the PICOS criteria. Subsequently, the full-text papers were assessed separately. When the two researchers disagreed on the potential eligibility of an article, a third researcher (W-SZ) was consulted for discussion and decision-making.

### Data extraction and coding

Two researchers (Y-HL and NX) independently extracted relevant data using a pre-designed sheet. The extracted information included reference details (first author), publication year, country, health status of participants, sample size (N), age, exercise characteristics (frequency, intensity, time, type, duration) of the experimental and control groups, and PWV indicators. The mean and standard deviation of each PWV indicator result were extracted. If the results were presented in graphical form, GetData Graph Digitizer software (version 2.26) was used for data extraction. When an included study reported multiple PWV indicators, all indicators were included and analyzed. If a study had two or more exercise groups, each was included separately and compared with the control group. When a study had two or more control groups, all control groups were included and compared with the exercise group.

The studies were coded based on their characteristics. Participant characteristics were coded as follows: (1) Mean age was coded as middle-aged (< 65 years) and elderly (≥ 65 years); (2) Health status of participants was coded as healthy, hypertensive, and with arterial disease. Exercise protocol was coded as follows: (1) Type of exercise was coded as aerobic exercise and resistance exercise; (2) Duration was coded as 8 weeks and 12 weeks; (3) Frequency was coded as < 3 times/week and ≥ 3 times/week. Four studies did not report exercise intensity, so no analysis was conducted for the exercise intensity moderator. Outcome measures were coded as follows: (1) cfPWV, baPWV, faPWV, aortic PWV, leg PWV, and hbPWV were combined into SAPWV for analysis; (2) PWV was coded based on the artery as central PWV (cfPWV and aortic PWV) and peripheral PWV (baPWV, faPWV, leg PWV, and hbPWV); (3) cfPWV, baPWV, faPWV, aortic PWV, leg PWV, and hbPWV were analyzed individually. Although SAPWV was aggregated from multiple PWV measures to assess systemic arterial stiffness, we prioritized subgroup analyses by anatomical site - central (cfPWV, aortic PWV) versus peripheral (baPWV, faPWV, hbPWV, leg PWV) - to ensure that the findings remained physiologically interpretable and not obscured by methodological aggregation.

### Assessment of study quality

Two researchers (F-FR and S-JM) independently assessed the quality of all included studies using the Physiotherapy Evidence Database (PEDro) scale. The PEDro scale consists of the following 11 items: (1) eligibility criteria; (2) random allocation; (3) concealed allocation; (4) baseline similarity; (5) subject blinding; (6) therapist blinding; (7) assessor blinding; (8) more than 85% retention; (9) intention-to-treat analysis; (10) between-group comparisons; (11) point and variability measures. Studies scoring above 5–8 were considered high quality (9–10 points: very high quality), those scoring 4 or 5 were considered moderate quality, and those scoring 0–3 were considered low quality. Any inconsistencies between the two researchers were discussed and resolved with a third researcher (W-SZ).

### Statistical analysis

Statistical analyses were conducted in R (version 4.4.0) using the *metafor Package* 3.0–2 [53, 54]. Studies included in the analysis contained two or more effect sizes (ESs) or compared multiple exercise groups with a single control group [33, 35, 36, 50]. In addition, several studies compared two or more PWV indicators [33–36]. To account for the dependency of ES within the same article, a three-level meta-analysis with random effects was employed. Three-level meta-analysis has been proven to be more effective in handling the dependency of ES than traditional univariate meta-analysis methods [55, 56]. Specifically, this method models three different sources of variance: the sampling variance in ES (modeled at level 1), the variance in ESs within studies (modeled at level 2), and the variance in ESs between studies (modeled at level 3) [56, 57]. A one-tailed likelihood ratio test was used to assess the statistical significance of the random variances at level 2 and level 3. The presence of statistically significant variance at any level indicates heterogeneity among the ESs. Statistical significance was defined as *p* < 0.05.

The sign and magnitude of the ES have specific interpretations. A negative ES indicates that, compared with the control group, exercise training has a significant beneficial effect on PWV. The magnitudes of the ES were categorized as follows: (1) small (g > −0.20), (2) small to medium (g = −0.20–−0.49), (3) medium (g = −0.5–−0.79), and (4) large (g ≤ −0.80) [58]. In addition, the total heterogeneity between studies was calculated using the I² statistic, where 1%−49%, 50%−74%, and 75%−100% represent low, moderate, and high heterogeneity, respectively [59]. To investigate publication bias, a funnel plot and a multilevel extension of Egger’s test were conducted [60]. Moreover, sensitivity analyses were conducted to assess the impact of outliers and influential studies on the pooled ES [61].

The specific procedure of the meta-analysis is as follows: (1) Estimating the overall effect of exercise training on SAPWV by pooling all PWV ESs; (2) Subgroup analyses were conducted on categorical variables including participant characteristics (age and health status), PWV sub-indicators (cfPWV, baPWV, faPWV, aortic PWV, leg PWV, and hbPWV), vascular anatomical sites (central PWV and peripheral PWV), and exercise characteristics (type of exercise, exercise frequency per week, and duration of exercise).

## Results

**Fig. 1:**
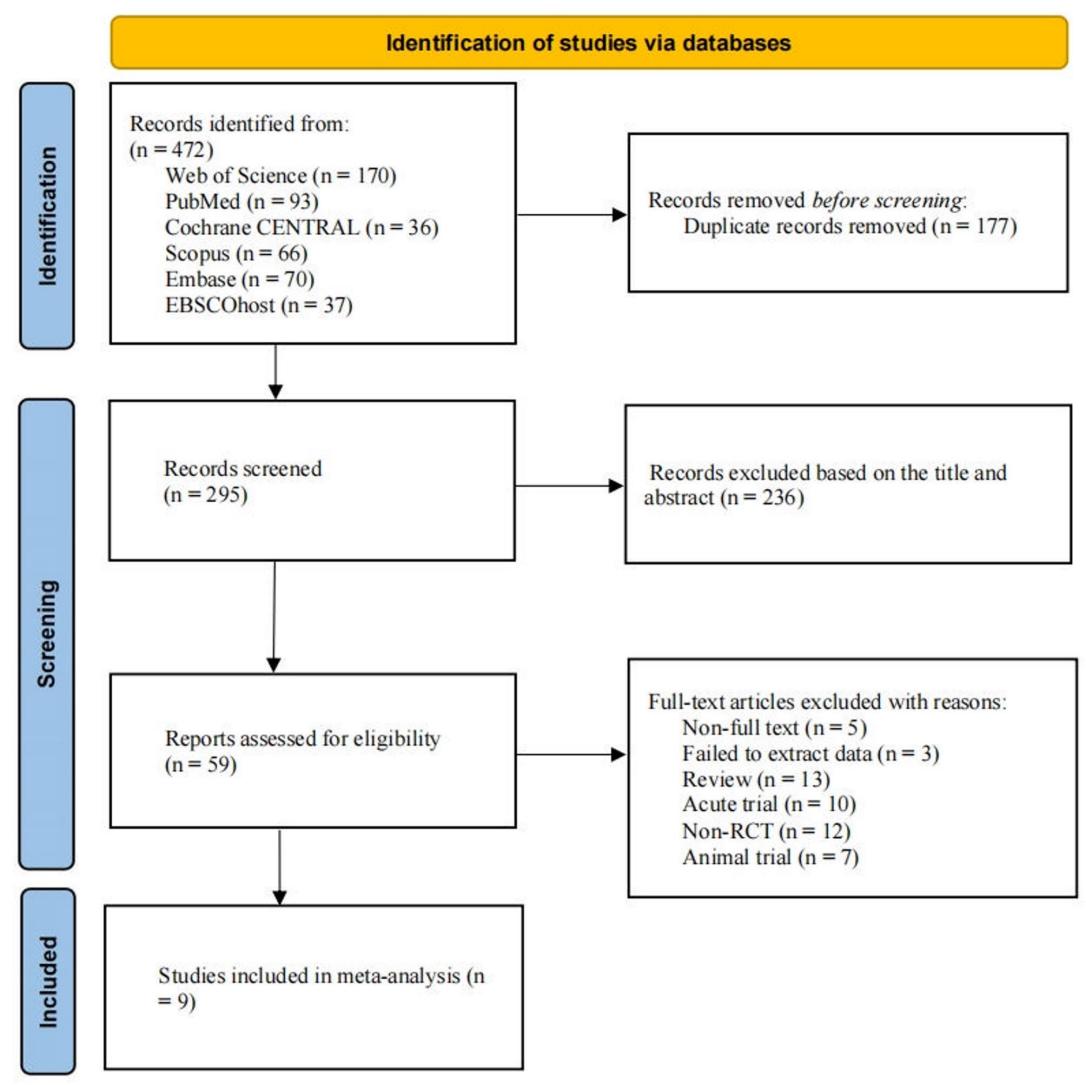
PRISMA flowchart of study selection. *n* number, *RCT* randomized controlled trial

Figure 1 illustrates the specific procedures for literature search and screening. A total of 472 articles were identified. After removing duplicates (*k* = 177) using EndNote reference management software, 295 articles proceeded to the title and abstract screening stage. In this process, 236 articles were excluded as they were deemed irrelevant to the research topic. Subsequently, the remaining 59 articles were subjected to full-text reading to evaluate the relevance further, and 50 articles that did not meet the inclusion criteria were excluded. Ultimately, nine articles met the inclusion criteria, including six articles identified through our database search that were also cited in Yang et al. (2024) and Zhou et al. (2023) [31, 32, 34–36, 50], along with three additional RCTs [33, 51, 52] found in the current search.

### Characteristics of included studies

Table 1 presents the nine studies included from 2012 to 2022. These studies were conducted in the following countries: the United States (*n* = 5), Japan (*n* = 3), and South Korea (*n* = 1). A total of 496 postmenopausal or older women with a mean age ranging from 55.5 to 74.09 years (270 in the exercise group and 226 in the control group) were included in the studies. Exercise frequency varied from 2 to 4 sessions per week. Five studies implemented low-to-high intensity exercise protocols, while one study implemented a low-intensity regimen in Exercise Group 1 with unreported intensity in Exercise Group 2. The remaining three studies did not specify exercise intensity. Exercise modalities included aerobic training (*n* = 5) and resistance training (*n* = 4), with session times lasting 20–90 min. The exercise duration is 8 weeks and 12 weeks. Regarding outcome measures, seven studies reported baPWV [31–36, 50]. Leg PWV was reported in three studies [34, 35, 52]. cfPWV was reported in three studies [33, 36, 51], while aortic pulse wave velocity (aorticPWV) was reported in two studies [34, 35]. Single studies reported femoral-ankle pulse wave velocity (faPWV) [36] and heart-brachial pulse wave velocity (hbPWV) [33].

**Table 1:** Characteristics of included studies (*k* = 9)

Included studies location	Health condition	Experimental group			Control group			Outcomes
		*N*	Ages (yrs)	Exercise program	*N*	Ages (yrs)	Exercise program	
Figueroa et al. (2014) [34] USA	HTN	13	55.5 ± 0.7	F: 3 times/wk I: no report Ti: 30–60 s/set, 6 sets Ty: whole-body vibration resistance exercise (four leg exercises, 25–40 Hz) D: 12 wks	12	56.4 ± 1	No exercise	baPWV leg PWV aorticPWV
Figueroa et al. (2015) [35] USA	high ankle SBP	12	56 ± 1	F: 3 times/wk I: no report Ti: 30–60 s/set, 6 sets Ty: whole-body vibration resistance exercise (four leg exercises, 25–40 Hz) D: 12 wks	12	58 ± 1	No exercise	baPWV leg PWV aorticPWV
	normal ankle SBP	12	58 ± 1					
Ha et al. (2018) [51] Korea	HLH	11	74.09 ± 4.21	F: 3 times/wk I: RPE9-14 or 30–60%HRR Ty: aquatic exercise Ti: 50 min D: 12 wks	8	76.00 ± 5.52	maintain regular activity	cfPWV
Jaime et al. (2019) [36] USA	HLH	12	64 ± 1	F: 3 times/wk I: 40%1RM, 15 repetitions Ty: low-intensity resistance exercise (four leg exercises, 2–3 set/exercise) Ti: 30–35 min D: 12 wks	8	67 ± 1	maintain regular activity	baPWV cfPWV faPWV
		12	64 ± 1	F: 3 times/wk I: no report Ty: whole-body vibration resistance exercise (four leg exercises, 24–40 Hz, 2–3 set/exercise) Ti: 30–35 min D: 12 wks				
Kobayashi et al. (2022) [33] Japan	HLH	15	62.5 ± 2.8	F: 2 times/wk I: 65%HRR Ty: aerobic exercise Ti: 30 min D: 8 wks	15	60.9 ± 3.9	no training	baPWV cfPWV hbPWV
		15	62.9 ± 1.9	F: 4 times/wk I: 65%HRR Ty: aerobic exercise Ti: 30 min D: 8 wks				
Miura et al. (2015) [50] Japan	HTN_stage 1	45	73.1 ± 6.0	F: 2 times/wk I: 15–20 repetitions Ty: circuit resistance exercise (6–8 exercises, 3–5 set/exercise) Ti: 90 min D: 12 wks	47	70.0 ± 6.9	maintain regular activity	baPWV
	HLH	55	72.5 ± 7.0		53	72.1 ± 5.3		
Ohta et al. (2012) [31] Japan	HLH	13	72.2 ± 4.2	F: 3 times/wk I: LA < 4 mmol/l and/or RPE < 17 Ty: bench step exercise Ti: 30–60 min/day, 140 min/wk D: 12 wks	13	71.5 ± 7.4	maintain an ordinary lifestyle	baPWV
Park et al.(2019) [52] USA	PAD	35	70.0 ± 10.0	F: 4 times/wk I: 50–85%HRR Ty: aquatic walking Ti: 60 min D: 12 wks	37	71.0 ± 8.0	no exercise	leg PWV
Wong et al. (2018) [32] USA	HTN_stage 2	20	59 ± 1	F: 4 times/wk I: RPE11-13 Ty: aerobic exercise (climbing 192 stair steps, 2–5 times/day) Ti: 20–40 min D: 12 wks	21	59 ± 1	no exercise	baPWV

*k* Number of included studies, *yrs* Years, *HTN* Hypertension, *HLH* Health, *PAD* Peripheral artery disease, *RA* Rheumatoid arthritis, *SBP* Systolic blood pressure, *F* Frequency, *I* Intensity, *Ti* Time, *Ty* Type, *D* Duration, *wk* Week, *wks* Weeks, *min* Minutes, *sec* Second, *HRR* Heart rate reserve, *baPWV* Brachial-ankle pulse wave velocity, *cfPWV* Carotid-femoral pulse wave velocity, *hbPWV* Heart-brachial pulse wave velocity, *faPWV* Femoral-ankle pulse wave velocity, *leg PWV* Leg pulse wave velocity, *aorticPWV* Aortic pulse wave velocity

### Methodological quality assessment

Table 2 presents the methodological quality assessment results of the nine included studies. Five studies were of good quality (≥ 6 points), and four studies were of moderate quality (4–5 points). The average score of the included studies was 5.67 points.

**Table 2:** Quality assessment of included studies (*k* = 9)

Author (year)	Year	A	B	C	D	E	F	G	H	I	J	K	Total score	Overall study quality
Figueroa et al. [34]	2014	1	1	0	1	0	0	1	0	0	1	1	5	Fair
Figueroa et al. [35]	2015	1	1	0	1	0	0	1	1	0	1	1	6	Good
Ha et al. [51]	2018	1	1	0	1	0	0	0	1	1	1	1	6	Fair
Jaime et al. [36]	2019	1	1	0	1	0	0	1	0	0	1	1	5	Fair
Kobayashi et al. [33]	2022	1	1	0	1	0	0	0	1	1	1	1	6	Fair
Miura et al. [50]	2015	1	1	0	1	0	0	0	1	0	1	1	5	Fair
Ohta et al. [31]	2012	1	1	0	1	0	0	0	1	1	1	1	6	Fair
Park et al. [52]	2019	1	1	0	1	0	0	1	1	1	1	1	7	Good
Wong et al. [32]	2018	1	1	0	1	0	0	0	1	0	1	1	5	Fair
Mean score and quality													5.67	Good

*k* number of included studies, *A* Eligibility criteria, *B* allocation of randomization, *C* Concealed allocation, *D* Similarity baseline, *E* Subject blinding, *F* Therapist blinding, *G* Assessor blinding, *H* more than 85% retention, *I* Intention-to-treat analysis, *J* Between-group comparisons, *K* Point and variability measures, *1* explicitly described and present in details, *0* absent, inadequately described, or unclear

### SAPWV

**Fig. 2:**
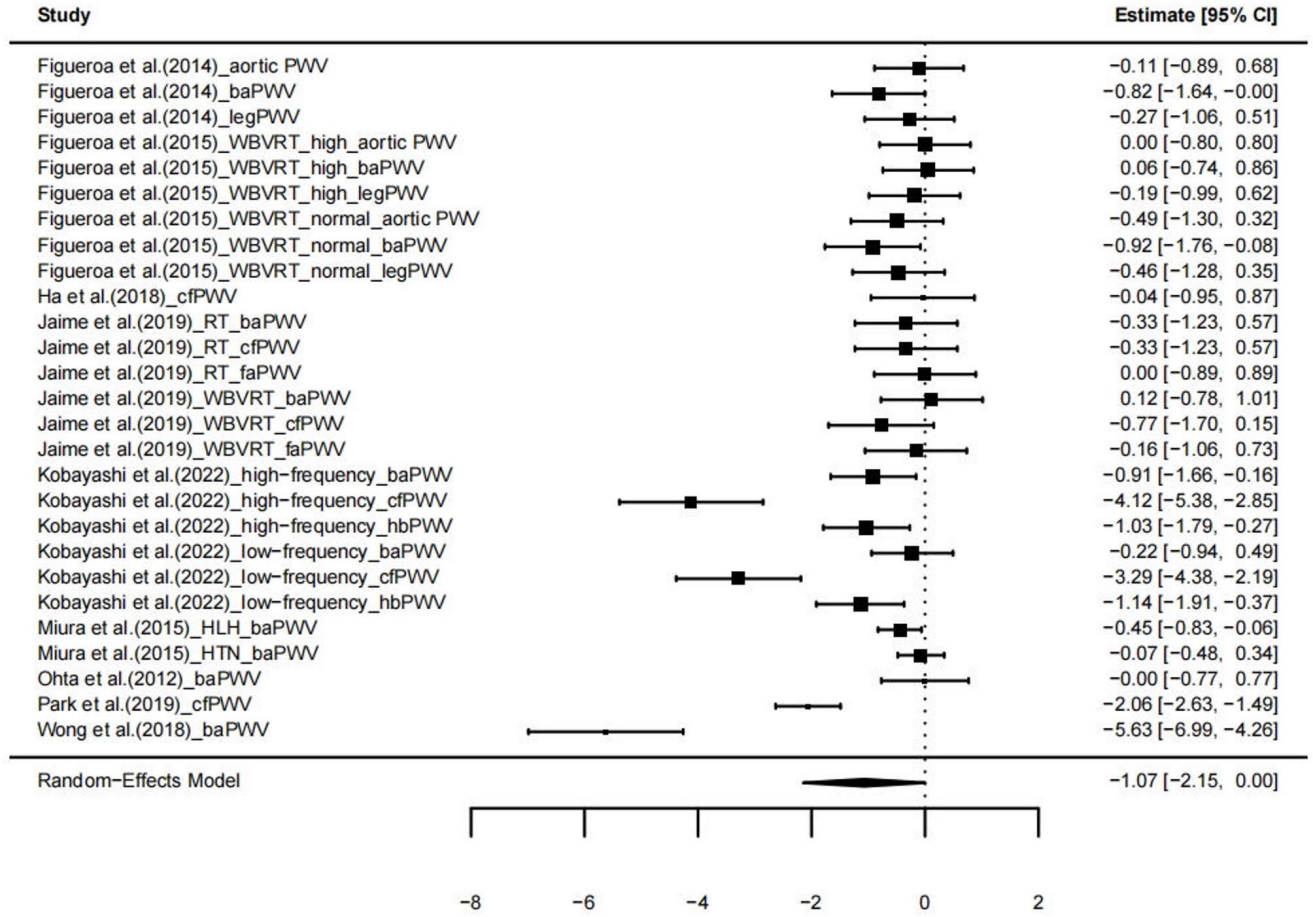
Forest plot of exercise on systemic arterial pulse wave velocity *PWV* Pulse wave velocity, *CI* Confidence intervals, *WBVRT* Whole-body vibration resistance exercise, *RT* Resistance exercise, *HLH* Health, *HTN* Hypertension

Compared with the control group, exercise training (k = 9, 27 ESs) had a marginally significant effect on SAPWV in postmenopausal women (g = −1.07, 95% CI = −2.15, 0.00; *p* = 0.05), but the confidence interval includes zero-indicating substantial uncertainty in the estimate. This suggests that the true effect may be null or even negative, and thus the evidence for a definitive benefit is limited (see Fig. 2). Level 2 heterogeneity was low (I² = 14.43%), level 3 heterogeneity was high (I² = 80.09%), and the total heterogeneity in ESs was extremely high (I² = 94.43%). This very high heterogeneity undermines the reliability of the pooled estimate. The true effect may vary significantly depending on study-specific factors such as participant characteristics, intervention protocols, and measurement methods. Consequently, moderator analyses were conducted to explore potential sources of heterogeneity.

### Moderator analyses

#### PWV Sub-indicators

The results of the subgroup analysis are presented in Table 3. Tests of moderation revealed that PWV sub-indicators were statistically significant moderators (F = 3.20, *p* = 0.022), indicating that the PWV sub-indicators significantly moderated the effects of exercise training on SAPWV in postmenopausal women. The subgroup results demonstrated that exercise significantly improved cfPWV (g = −2.44; 95% CI = −3.94, −0.93; *p* = 0.003), but did not significantly affect aortic PWV (g = −0.54; 95% CI = −2.09, 1.00; *p* = 0.476), baPWV (g = −0.88; 95% CI = −2.23, 0.47; *p* = 0.191), faPWV (g = −1.37; 95% CI = −3.07, 0.33; *p* = 0.108), hbPWV (g = −0.86; 95% CI = −2.51, 0.79; *p* = 0.292), or leg PWV (g = −0.69; 95% CI = −2.20, 0.82; *p* = 0.353).

**Table 3:** Subgroup analyses of exercise on systemic arterial pulse wave velocity

Subgroup analysis	k	Hedges’g (95%CI)	*p* value	Test of Moderation
PWV Sub-indicators				*F* (6, 21) = 3.20, *p =* 0.022
aortic PWV	3	−0.54 (−2.09, 1.00)	0.476	
baPWV	11	−0.88 (−2.23, 0.47)	0.191	
cfPWV	5	**−2.44 (−3.94**,** −0.93)**	**0.003**	
faPWV	2	−1.37 (−3.07, 0.33)	0.108	
hbPWV	2	−0.86 (−2.51, 0.79)	0.292	
leg PWV	4	−0.69 (−2.20, 0.82)	0.353	
Regional PWV				*F* (2, 25) = 3.83, *p* = 0.036
Central PWV	8	**−1.57 (−2.84**,** −0.30)**	**0.018**	
Peripheral PWV	19	−0.93 (−2.11, 0.26)	0.12	
Age				*F* (2, 25) = 2.99, *p* = 0.068
< 65 years	23	**−1.52 (−2.80 −0.24)**	**0.022**	
≥ 65 years	4	−0.11 (−1.98, 1.77)	0.906	
Health condition				*F* (3, 24) = 1.44, *p* = 0.256
Health	15	−0.86 (−2.24, 0.53)	0.214	
Hypertension	11	−1.10 (−2.60, 0.40)	0.142	
Arterial disease	1	−2.06 (−5.57, 1.45)	0.237	
Exercise type				*F* (2, 25) = 3.65, *p* = 0.041
Aerobic exercise	10	**−1.76 (−3.13**,** −0.40)**	**0.011**	
Strength exercise	17	−0.31 (−1.71, 1.09)	0.654	
Exercise frequency				*F* (2, 25) = 2.31, *p* = 0.120
< 3 sessions/wk	5	−0.75 (−2.22, 0.72)	0.305	
≥ 3 sessions/wk	22	**−1.14 (−2.25**,** −0.04)**	**0.043**	
Exercise duration				*F* (2, 25) = 1.87, *p* = 0.175
8 weeks	6	−1.57 (−4.94, 1.80)	0.347	
12 weeks	21	−1.02 (−2.27, 0.23)	0.105	

*k* number of included studies, *ES* effect size, *CI* confidence interval, *Central PWV* aorta artery and carotid-femoral artery, *Peripheral PWV* brachial-ankle artery, femoral-ankle artery, heart-brachial artery, and leg artery

*p* < 0.05 indicates statistical significance

#### Regional PWV

The results of the moderators’ test revealed that regional PWV was a significant moderator (F = 3.83, *p* = 0.036), indicating that the regional PWV significantly moderated the effect of exercise training on SAPWV in postmenopausal women (see Table 3**)**. The results demonstrated that exercise significantly improved central PWV (g = −1.57; 95% CI = −2.84, −0.30; *p* = 0.018), but did not affect peripheral PWV (g = −1.57; 95% CI = −2.11, 0.26; *p* = 0.12).

#### Exercise characteristics

In the subgroup analysis of exercise characteristics, neither the exercise duration (F = 1.87, *p* = 0.175) nor the exercise frequency (F = 2.31, *p* = 0.120) was a significant moderator, whereas the exercise type was a significant moderator (F = 3.65, *p* = 0.041) (see Table 3). The results for exercise type indicated that aerobic exercise had a significant effect on SAPWV (g = −1.76; 95% CI = −3.13, −0.40; *p* = 0.011), while resistance training did not significant effect on SAPWV (g = −0.31; 95% CI = −1.71, 1.09; *p* = 0.654). The results for exercise frequency indicated that ≥ 3 sessions/wk had a significant effect on SAPWV (g = −1.14; 95% CI = −2.25, −0.04; *p* = 0.043), while < 3 sessions/wk did not have a significant effect on SAPWV (g = −0.75; 95% CI = −2.22, 0.72; *p* = 0.305). The results for exercise duration indicated that neither 8 weeks (g = −1.57; 95% CI = −4.94, 1.80; *p* = 0.347) nor 12 weeks (g = −1.02; 95% CI = −2.27, 0.23; *p* = 0.105) had a significant effect on SAPWV.

#### Sample characteristics

Regarding sample characteristics (see Table 3), neither age (F = 2.99, *p* = 0.068) nor health condition was a significant moderator (F = 1.44, *p* = 0.256). The results for the age subgroup indicated that < 65 years had a significant effect on SAPWV (g = −1.52; 95% CI = −2.80, −0.24; *p* = 0.022), while ≥ 65 years did not have a significant effect on SAPWV (g = −0.11; 95% CI = −1.98, 1.77; *p* = 0.906). The results for health condition indicated that neither Health (g = −0.86; 95% CI = −2.24, 0.53; *p* = 0.214), Hypertension (g = −1.10; 95% CI = −2.60, 0.40; *p* = 0.142), nor with Arterial disease (g = −2.06; 95% CI = −5.57, 1.45; *p* = 0.237) had a significant effect on SAPWV.

#### Sensitivity analysis

To investigate the impact of outliers and influential studies on the overall pooled ES, a sensitivity analysis was conducted. One study that assessed baPWV after a 12-week aerobic exercise intervention had a residual value exceeding the mean by three standard deviations [32]. After excluding this study, the overall effect of exercise on SAPWV was reduced by 0.45 (g = −0.62; 95% CI = −1.30, −0.11; *p* = 0.019). Further analysis was conducted by calculating Cook’s distance and determining whether its value exceeded three times the mean. It was found that the potential outlier was still the study by Wong et al. (2018). After excluding the outlier, the overall effect of exercise on SAPWV remained the same (g = −0.62; 95% CI = −1.30, −0.11; *p* = *0*.019).

#### Publication bias

**Fig. 3:**
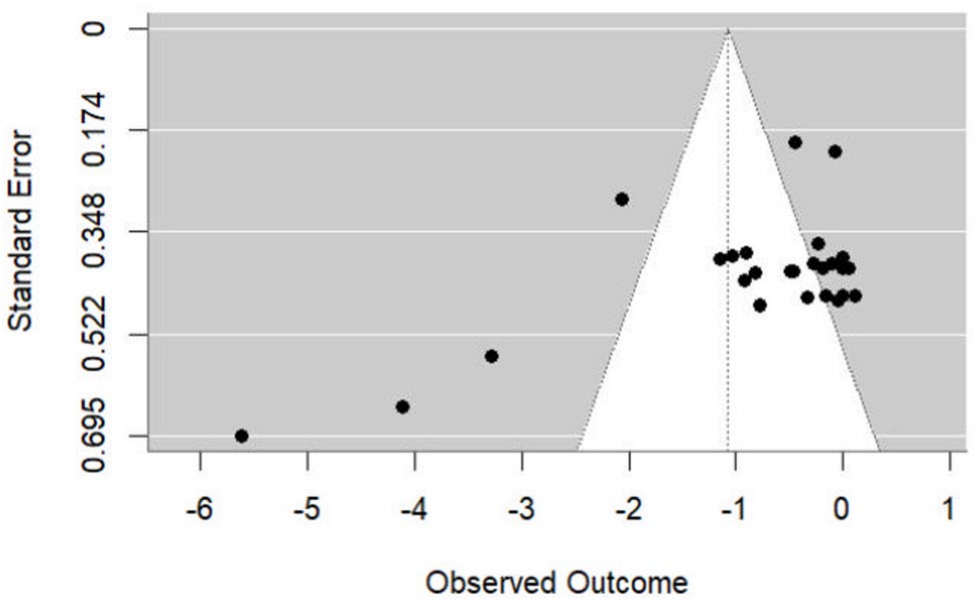
Funnel plot of potential publication bias

Based on visual examination, the funnel plot showed asymmetry of ESs, indicating the possibility of publication bias (see Fig. 3). The multilevel extension of Egger’s test revealed significant evidence of publication bias among the included studies [F (1,75) = 36.80, *p* < 0.0001].

## Discussion

The effect of exercise on SAPWV in postmenopausal women remains uncertain, as the overall pooled effect was only marginally significant (g = −1.07, 95% CI = −2.15, 0.00; *p* = 0.05), with the confidence interval including zero-indicating that the true effect could be null or even negative. This suggests that while there is a potential trend toward improvement, current evidence does not provide strong statistical support for a definitive benefit. Nevertheless, subgroup analyses revealed more robust and statistically significant improvements in specific subgroups, particularly those involving central arterial stiffness indicators such as cfPWV and central PWV. These findings should be interpreted cautiously, recognizing that they may reflect site-specific effects rather than a universal improvement in systemic arterial elasticity.

### SAPWV

SAPWV reflects systemic arterial elasticity and enables early detection of malignant changes in the vascular wall, holding significant implications for preventing vascular diseases [10]. We comprehensively analyzed all the arterial stiffness indicators included in the studies, and the results indicated that exercise had marginal significance in reducing SAPWV (g = −1.07, *p* = 0.05) compared with the control group, suggesting a potential trend toward SAPWV reduction and arterial elasticity improvement, though current statistical evidence remains insufficient. These findings align with recent meta-analytic results reported by Yang et al. (2024), which included six RCTs involving 243 hypertensive postmenopausal women and reported a significant 0.69 m/s reduction in baPWV [37].In addition, our previous meta-analysis of 19 RCTs (*n* = 466) indicated that exercise reduced PWV (including cfPWV, faPWV, and baPWV) by 0.79 m/s in postmenopausal women [38]. The present study updates and validates the findings of our previous study. Building on previous research, this study further updated and expanded the evidence base by including 27 RCTs with a total of 496 participants. The increased sample size and effect size enhance the reliability and credibility of the findings. The improvement in arterial elasticity is mainly due to the multiple positive effects of exercise training on blood vessels, including increased arterial blood flow perfusion due to exercise, which stimulates endothelial cells to upregulate the expression of eNOS [62], increased NO production [63, 64], and vascular endothelial structural remodeling and reduced smooth muscle tension [65, 66]. In addition, long-term regular exercise reduces sympathetic nerve excitability, up-regulates parasympathetic nerve activity, and optimizes vascular dilation function [67]. Long-term exercise can improve oxidative stress levels, increase arterial elastin, reduce collagen, enhance antioxidant effects, and improve endothelial-dependent vasodilation [64, 65], thereby improving arterial elasticity.

In summary, although the current study found that the effect of exercise intervention on SAPWV in postmenopausal women was only marginally significant, existing evidence and biological mechanisms suggest that exercise may indeed have a positive effect on improving arterial elasticity. Future research should further expand the sample size, optimize exercise intervention protocols, and employ more sensitive measurement indicators to clarify the effectiveness of exercise in reducing arterial stiffness. Furthermore, the high heterogeneity (I² = 94.43%) and the presence of publication bias (*p* < 0.0001) suggest that the observed effect may be overestimated. The sensitivity analysis revealed that the overall effect was largely driven by one study (Wong et al., 2018), which reported an unusually large effect size (g = −2.44). When this study was excluded, the effect remained significant but smaller (g = −0.62), indicating that the result is sensitive to outliers. These findings highlight the need for caution in drawing firm conclusions from the current evidence base.

### SAPWV subdomains and sample characteristics

The present study indicated that SAPWV subdomains significantly moderated the effect of exercise training on SAPWV in postmenopausal women. The results demonstrated that exercise training significantly improved cfPWV (g = −2.44, *p* = 0.003), but did not significantly improve aortic PWV, baPWV, faPWV, hbPWV, or leg PWV. Our results are consistent with a previous meta-analysis [68], which found that exercise significantly reduced cfPWV by 0.45 m/s in patients with cardiovascular disease. The greater improvement observed in our study may be related to the health condition of the participants, as the majority in our study were healthy, with only a few diagnosed with hypertension. In contrast, the participants in their study had cardiovascular disease and poorer arterial status, resulting in smaller improvements in arterial elasticity due to exercise. Zhang et al. pointed out that there were no significant improvements in the secretion of PGI_2_ and TXA_2_, which induce vasodilation [68]. It is worth noting that our study found that aerobic exercise significantly improved SAPWV, while resistance training did not. The study by Zhang et al. (2018) also supported the ineffectiveness of resistance training, with aerobic exercise reducing cfPWV by 0.42 m/s, while the resistance training showed no improvement (MD = −0.26, 95% CI: −0.72, 0.20, *p* = 0.27), suggesting that aerobic exercise may be preferential for improving arterial stiffness. The meta-analysis by Zaman et al. (2024) also supports our findings, indicating that 12–20 weeks of aerobic exercise training can significantly reduce arterial stiffness in hypertensive women, while resistance training has a smaller effect [69]. Previous studies have shown that continuous low-intensity exercise can effectively enhance arterial elasticity [70]. Aerobic exercise fits the characteristics of continuous low-intensity exercise [71]. The American College of Sports Medicine recommends aerobic exercise as a way to promote vascular health [51, 72]. The present study did not find that age or health status significantly moderated the effect on SAPWV. Exercise intervention only showed an improvement effect on SAPWV in the < 65 years group, but not in the ≥ 65 years group. In our study, 23 RCTs were included in the < 65 years group, while only 4 RCTs (from 3 articles, all showing no significant effect on PWV) were included in the ≥ 65 years group [31, 50, 51]. The small sample size in the ≥ 65 years subgroup may be the main reason for the lack of significant effect. It may also be related to health status. Compared with younger older women, older women have more cardiovascular health issues, such as hypertension, and lower cardiovascular health levels may result in less improvement in arterial elasticity due to exercise than in younger populations. A study by Miura et al. (2015) showed that exercise training had less effect on arterial stiffness in elderly hypertensive women than in elderly healthy women [50]. In addition, These adaptive responses may be attenuated in postmenopausal women due to the decline in estrogen levels with aging. Estrogen plays a crucial role in maintaining endothelial function by enhancing eNOS expression, reducing oxidative stress, and inhibiting smooth muscle proliferation [16, 73, 74]. The loss of estrogen after menopause diminishes the responsiveness of the vascular system to hemodynamic stimuli [75], potentially limiting the magnitude of improvement in SAPWV even with regular exercise. Therefore, while exercise remains an effective non-pharmacological strategy, its efficacy may be blunted in older, estrogen-deficient populations [76].

It is crucial to distinguish between different PWV indices when interpreting results. While both cfPWV and baPWV are used to assess arterial stiffness, they reflect distinct vascular segments with different physiological implications. cfPWV primarily measures stiffness in the central arteries (aorta and large proximal vessels), which are directly linked to left ventricular afterload and cardiovascular risk. In contrast, baPWV reflects stiffness in peripheral arteries, which may be influenced by local factors such as muscle mass, ankle joint mobility, and calcification. Therefore, the significant reduction in cfPWV observed in our study (g = −2.44) has stronger prognostic value than the non-significant changes in baPWV (g = −0.88). This distinction underscores the importance of analyzing central and peripheral PWV separately in future research.

### Characteristics of exercise

Identifying effective exercise characteristics is crucial for developing targeted exercise prescriptions to improve cardiovascular health. This study examined three characteristics of exercise training programs: intervention type, frequency, and duration. The study results indicated that aerobic exercise significantly reduced SAPWV, while resistance training had no effect. A training frequency of ≥ three sessions per week significantly decreased SAPWV, while < 3 times per week had no benefit. The study by Ashor et al. (2014) supports our findings, indicating that aerobic exercise significantly reduced SAPWV (g = −1.76, *p* = 0.011) [77]. These benefits may be attributed to hemodynamic improvements induced by aerobic training [77]. The beneficial effects of aerobic exercise on SAPWV are primarily mediated by enhanced endothelial shear stress, which upregulates eNOS activity, increases NO bioavailability, and promotes vasodilation and arterial compliance. These mechanisms directly reduce arterial stiffness and improve SAPWV [78]. In contrast, resistance training failed to elicit similar improvements, potentially due to insufficient hemodynamic stimulation from short-duration, high-intensity muscle contractions, resulting in limited arterial compliance enhancement [78]. High-intensity resistance training may also induce transient blood pressure elevation, increasing mechanical load on arterial walls and contributing to greater central arterial stiffness [79, 80]. A systematic review and meta-analysis by Miyachi (2013) further supports our findings [39], indicating no significant PWV improvement with moderate-intensity resistance training, while high-intensity resistance training increased arterial stiffness. However, a previous study by Zhou et al. (2023) contradicted these results [38], demonstrating that resistance exercise improved PWV in postmenopausal women. Notably, all RCTs included in Zhou et al. utilized low-to-moderate intensity resistance training. Collectively, these studies suggest that higher-intensity resistance exercise is more likely to increase arterial stiffness [38, 39, 77]. No subgroup analysis based on exercise intensity was conducted in the present study, as several included RCTs did not accurately report training intensity parameters.

Regular and frequent exercise stimuli are essential for improving arterial compliance. This study found that a weekly training frequency of ≥ 3 sessions significantly reduced SAPWV (g = −1.14, *p* = 0.043), whereas frequencies below this threshold showed no significant changes. This suggests the existence of a dose-threshold effect for exercise frequency. Ho et al. (2024) indicated that exercising only 1–2 times weekly fails to induce vascular adaptive remodeling [81]. Engaging in physical exercise for at least 3 days per week or accumulating 150 min of moderate-intensity exercise weekly is considered fundamental for cardiovascular health protection [82]. The American College of Sports Medicine (ACSM) recommends that adults perform moderate-to-vigorous aerobic exercise ≥ 3 days/week or moderate-intensity aerobic exercise ≥ 5 days/week to optimize cardiopulmonary and vascular health [83]. Insufficient exercise frequency may compromise vascular benefits; even with high-intensity single sessions, prolonged intervals between bouts can diminish training-induced adaptations and decrease cumulative adaptive effects [84]. Aerobic exercise performed ≥ 3 times weekly maintains arteries in a cycle of repeated stimulation and repair, progressively reducing arterial stiffness. Therefore, achieving a minimum weekly frequency of 3 sessions constitutes a critical prerequisite for improving arterial stiffness in postmenopausal women. Exercise intervention programs should prioritize sustaining adequate weekly training frequency to induce beneficial functional and structural vascular adaptations consistently.

### Strengths and limitations

Compared with previous meta-analyses [37, 38], the present study is the first to use a three-level meta-analysis to examine the effects of exercise on SAPWV, multiple PWV indicators, and central versus peripheral PWV in postmenopausal women. Our study also investigated several moderator variables, including age, health status, and exercise protocols, and identified critical information such as effective aerobic exercise modalities and a minimum frequency of ≥ 3 sessions/week for improving SAPWV. However, several limitations warrant consideration. First, despite comprehensive systematic searches across multiple databases, relevant studies may have been omitted. Second, despite our efforts to standardize the inclusion criteria, there may still be significant differences among studies in terms of participant baseline characteristics, specific implementation details of the intervention protocols, and measurement methods and timing of outcome indicators, which may lead to instability in the final analysis results. Third, a limitation is the absence of exercise intensity data in several included RCTs (e.g., heart rate reserve, RPE), preventing a formal moderator analysis and limiting our ability to assess how training intensity influences arterial stiffness improvements. Fourth, although SAPWV provides a comprehensive assessment of arterial stiffness, its aggregation of multiple PWV indices may obscure site-specific responses to exercise. Future studies should analyze central and peripheral PWV separately to clarify regional vascular adaptations. Fifth, while the subgroup analyses revealed significant improvements in cfPWV, central PWV, aerobic exercise, participants aged < 65 years, and those exercising ≥ 3 sessions per week, these findings should be interpreted as exploratory due to the relatively small number of included studies and the increased risk of Type I error. They serve as hypotheses for future research rather than definitive evidence. Sixth, among the 27 RCTs included, none conducted follow-up assessments. Therefore, we did not analyze follow-up results, and future studies should focus on investigating the duration of the benefits of exercise interventions on SAPWV. Seventh, a notable limitation is the absence of adverse event reporting across all included studies. No study reported details on exercise-related side effects such as musculoskeletal injuries, joint pain, or cardiovascular events during or after intervention. This lack of safety data limits our ability to assess the risk-benefit profile of exercise interventions in postmenopausal women, particularly regarding high-intensity resistance training, which may transiently elevate blood pressure and increase mechanical stress on arterial walls. Eighth, this study did not consider other lifestyle factors and mental health status [85], such as life’s essential 8, which may influence metabolic health and aging process. Moreover, there is also evidence that environmental factors such as air pollution may also affect frailty and circulation system [86, 87]. Ninth, the generalizability of our findings should be interpreted with caution, given the relatively limited number of eligible studies in this field and the constraints in fully exploring sources of heterogeneity. Furthermore, the methodological quality of several included RCTs was moderate, with an average PEDro score of 5.67, indicating a moderate overall evidence base. This suggests that potential biases may have influenced the results and reduced their reliability. As such, the strength of our conclusions regarding the efficacy of exercise interventions on arterial stiffness in postmenopausal women should be tempered. Final, a key limitation is the underrepresentation of older postmenopausal women (aged ≥ 65 years), with only four RCTs included in this subgroup. This limits the generalizability of our findings to this population. Furthermore, several studies relied on age alone to infer menopause status rather than objective confirmation (e.g., amenorrhea duration, hormone levels). Although we conducted a sensitivity analysis excluding these studies and found consistent results, caution is still warranted when applying our conclusions to women aged ≥ 65 years or those without confirmed menopause.

## Conclusions

This systematic review and meta-analysis confirms that the overall effect of exercise on SAPWV was marginally significant, indicating a potential trend toward improvement but with considerable uncertainty. More pronounced effects were observed in specific subgroups and exercise types. Specifically, exercise significantly improved cfPWV and central PWV. Furthermore, significant improvements were observed in participants aged < 65 years, those exercising ≥ 3 times per week, and those engaging in aerobic exercise. These findings suggest that the benefits of exercise on arterial stiffness are not uniform and are influenced by key factors, including age, exercise modality, and frequency. However, the high heterogeneity and marginal significance of the overall effect underscore the need for caution in interpreting these results. Future large-scale, high-quality RCTs are essential to validate these findings and to determine the optimal exercise prescription for improving arterial elasticity in postmenopausal women.

## Supplementary Information

Supplementary Material 1.

## Data Availability

All authors confirm that the data supporting the findings of the present study are available within the article [and/or] its supplementary materials.

## Abbreviations

SAPWV: Systemic arterial pulse wave velocity
PWV: Pulse wave velocity
cfPWV: Carotid-femoral pulse wave velocity
baPWV: Brachial-ankle pulse wave velocity
faPWV: Femoral-ankle pulse wave velocity
hbPWV: Heart-brachial pulse wave velocity
RCTs: Randomized controlled trials
PEDro: Physiotherapy evidence database
HRT: Hormone replacement therapy
ESs: Effect sizes
CI: Confidence intervals
