# Supplementary material for: Effects of exercise training on systemic arterial pulse wave velocity in postmenopausal women: an updated systematic review and meta-analysis of randomized controlled trials
Source: BMC Sports Sci Med Rehabil. 2025 Nov 11;17:329. doi: 10.1186/s13102-025-01382-1 (PMC12606885; doi:10.1186/s13102-025-01382-1)
Supplement: Supplementary file 1 — Supplementary Material 1. [file 13102_2025_1382_MOESM1_ESM.docx]

**Supplementary Material:** Database search strategies.

| **Database** | **Search strategies** | **Results** |
| --- | --- | --- |
| Embase | #1: Postmenopause OR Postmenopausal OR Menopause OR Menopausal OR "Postmenopausal women" OR "Middle-aged and elderly women" OR "Elderly women" OR "Aged women" OR "Senior women"  #2: "Aerobic exercise" OR "Aerobic training" OR "Resistance exercise" OR "Resistance training" OR "Resistance program" OR "Strength exercise" OR "Strength training" OR "Weight-lifting" OR "Weight-lifting exercise"  #3: "Arterial stiffness" OR "Arterial thickness" OR "Arterial elasticity" OR "Arterial compliance" OR "Vascular stiffness" OR "Aortic stiffness" OR "Pulse wave velocity" OR "PWV" OR "Aortic pulse wave velocity" OR "Aortic PWV" OR "brachial ankle pulse wave velocity" OR "baPWV" OR "carotid femoral pulse wave velocity" OR "cfPWV" OR "femoral ankle pulse wave velocity" OR "faPWV" OR "heart brachial pulse wave velocity" OR "hbPWV" OR "leg pulse wave velocity" OR "legPWV"  #1 AND #2 AND #3  Search date: April 8th, 2025. | 70 |
| EBSCOhost | #1: (Postmenopause OR Postmenopausal OR Menopause OR Menopausal OR "Postmenopausal women" OR "Middle-aged and elderly women" OR "Elderly women" OR "Aged women" OR "Senior women").ti,ab  #2: ("Aerobic exercise" OR "Aerobic training" OR "Resistance exercise" OR "Resistance training" OR "Resistance program" OR "Strength exercise" OR "Strength training" OR "Weight-lifting" OR "Weight-lifting exercise").ti,ab  #3: ("Arterial stiffness" OR "Arterial thickness" OR "Arterial elasticity" OR "Arterial compliance" OR "Vascular stiffness" OR "Aortic stiffness" OR "Pulse wave velocity" OR "PWV" OR "Aortic pulse wave velocity" OR "Aortic PWV" OR "brachial ankle pulse wave velocity" OR "baPWV" OR "carotid femoral pulse wave velocity" OR "cfPWV" OR "femoral ankle pulse wave velocity" OR "faPWV" OR "heart brachial pulse wave velocity" OR "hbPWV" OR "leg pulse wave velocity" OR "legPWV").ti,ab  #1 AND #2 AND #3  Search date: April 8th, 2025. | 37 |
| Scopus | #1: Title, Abstract, Keywords: (Postmenopause OR Postmenopausal OR Menopause OR Menopausal OR "Postmenopausal women" OR "Middle-aged and elderly women" OR "Elderly women" OR "Aged women" OR "Senior women")  #2: Title, Abstract, Keywords: ("Aerobic exercise" OR "Aerobic training" OR "Resistance exercise" OR "Resistance training" OR "Resistance program" OR "Strength exercise" OR "Strength training" OR "Weight-lifting" OR "Weight-lifting exercise")  #3: Title, Abstract, Keywords: ("Arterial stiffness" OR "Arterial thickness" OR "Arterial elasticity" OR "Arterial compliance" OR "Vascular stiffness" OR "Aortic stiffness" OR "Pulse wave velocity" OR "PWV" OR "Aortic pulse wave velocity" OR "Aortic PWV" OR "brachial ankle pulse wave velocity" OR "baPWV" OR "carotid femoral pulse wave velocity" OR "cfPWV" OR "femoral ankle pulse wave velocity" OR "faPWV" OR "heart brachial pulse wave velocity" OR "hbPWV" OR "leg pulse wave velocity" OR "legPWV")  #1 AND #2 AND #3  Search date: April 8th, 2025. | 66 |
| Web of Science | #1: TS= (Postmenopause OR Postmenopausal OR Menopause OR Menopausal OR "Postmenopausal women" OR "Middle-aged and elderly women" OR "Elderly women" OR "Aged women" OR "Senior women")  #2: TS= ("Aerobic exercise" OR "Aerobic training" OR "Resistance exercise" OR "Resistance training" OR "Resistance program" OR "Strength exercise" OR "Strength training" OR "Weight-lifting" OR "Weight-lifting exercise")  #3: TS= ("Arterial stiffness" OR "Arterial thickness" OR "Arterial elasticity" OR "Arterial compliance" OR "Vascular stiffness" OR "Aortic stiffness" OR "Pulse wave velocity" OR "PWV" OR "Aortic pulse wave velocity" OR "Aortic PWV" OR "brachial ankle pulse wave velocity" OR "baPWV" OR "carotid femoral pulse wave velocity" OR "cfPWV" OR "femoral ankle pulse wave velocity" OR "faPWV" OR "heart brachial pulse wave velocity" OR "hbPWV" OR "leg pulse wave velocity" OR "legPWV" ).  #1 AND #2 AND #3  Search date: April 8th, 2025. | 170 |
| PubMed | ("Postmenopause"[Title/Abstract] OR "Postmenopausal"[Title/Abstract] OR "Menopause"[Title/Abstract] OR "Menopausal"[Title/Abstract] OR "postmenopausal women"[Title/Abstract] OR ("Middle-aged"[Title/Abstract] OR "elderly women"[Title/Abstract]) OR "elderly women"[Title/Abstract] OR "aged women"[Title/Abstract] OR "senior women"[Title/Abstract]) AND ("aerobic exercise"[Title/Abstract] OR "aerobic training"[Title/Abstract] OR "resistance exercise"[Title/Abstract] OR "resistance training"[Title/Abstract] OR "resistance program"[Title/Abstract] OR "strength exercise"[Title/Abstract] OR "strength training"[Title/Abstract] OR "Weight-lifting"[Title/Abstract] OR "weight lifting exercise"[Title/Abstract]) AND ("arterial stiffness"[Title/Abstract] OR "arterial thickness"[Title/Abstract] OR "arterial elasticity"[Title/Abstract] OR "arterial compliance"[Title/Abstract] OR "vascular stiffness"[Title/Abstract] OR "aortic stiffness"[Title/Abstract] OR "pulse wave velocity"[Title/Abstract] OR "PWV"[Title/Abstract] OR "Aortic pulse wave velocity"[Title/Abstract] OR "Aortic PWV"[Title/Abstract] OR "brachial ankle pulse wave velocity"[Title/Abstract] OR "baPWV"[Title/Abstract] OR "carotid femoral pulse wave velocity"[Title/Abstract] OR "cfPWV"[Title/Abstract] OR "femoral ankle pulse wave velocity"[Title/Abstract] OR "faPWV"[Title/Abstract] OR "heart brachial pulse wave velocity"[Title/Abstract] OR "hbPWV"[Title/Abstract] OR "leg pulse wave velocity"[Title/Abstract] OR "legPWV"[Title/Abstract]).  Search date: April 8th, 2025. | 93 |
| Cochrane CENTRAL | #1: (Postmenopause OR Postmenopausal OR Menopause OR Menopausal OR "Postmenopausal women" OR "Middle-aged and elderly women" OR "Elderly women" OR "Aged women" OR "Senior women").title,abstract,keyword  #2: ("Aerobic exercise" OR "Aerobic training" OR "Resistance exercise" OR "Resistance training" OR "Resistance program" OR "Strength exercise" OR "Strength training" OR "Weight-lifting" OR "Weight-lifting exercise").title,abstract,keyword  #3: ("Arterial stiffness" OR "Arterial thickness" OR "Arterial elasticity" OR "Arterial compliance" OR "Vascular stiffness" OR "Aortic stiffness" OR "Pulse wave velocity" OR "PWV" OR "Aortic pulse wave velocity" OR "Aortic PWV" OR "brachial ankle pulse wave velocity" OR "baPWV" OR "carotid femoral pulse wave velocity" OR "cfPWV" OR "femoral ankle pulse wave velocity" OR "faPWV" OR "heart brachial pulse wave velocity" OR "hbPWV" OR "leg pulse wave velocity" OR "legPWV").title,abstract,keyword  #1 AND #2 AND #3  Search date: April 8th, 2025. | 36 |
